# Effect of Conventional Cataract Surgery and Femtosecond Laser-Assisted Cataract Surgery on Bruch's Membrane Opening-Minimum Rim Width, Retinal Nerve Fiber Layer, and Macular Thickness

**DOI:** 10.1155/2023/8345333

**Published:** 2023-02-07

**Authors:** Josefina Reñones, Alfonso Anton, Jesús María Gonzalez-Martin, Humberto Carreras, Juan Francisco Loro-Ferrer

**Affiliations:** ^1^Vithas Eurocanarias Instituto Oftalmológico, Las Palmas de Gran Canaria 35004, Spain; ^2^Universidad de Las Palmas de Gran Canaria (ULPGC), Las Palmas de Gran Canaria 35001, Spain; ^3^Universidad Internacional de Cataluña (UIC), Barcelona 08195, Spain; ^4^Institut Català de Retina (ICR), Barcelona 08017, Spain; ^5^Department of Research, Hospital Universitario de Gran Canaria Doctor Negrín, Las Palmas de Gran Canaria 35019, Spain

## Abstract

**Purpose:**

To evaluate the effect of conventional cataract surgery (CCS) and femtosecond laser-assisted cataract surgery (FLACS) on Bruch's membrane opening-minimum rim width (BMO-MRW), peripapillary retinal nerve fiber layer thickness (RNFL), and macular thickness (MT) using spectral-domain optical coherence tomography (SD-OCT).

**Methods:**

BMO-MRW, RNFL, and MT were measured using SD-OCT preoperatively, 1 month and 6 months after surgery in both CCS and FLACS groups. Differences between preoperative and postoperative values were evaluated in both groups. The postoperative changes were evaluated in each group and compared between groups.

**Results:**

A total of 146 eyes of 146 patients were included in this study, 65 underwent CCS, and 81 underwent FLACS. One month after surgery, there was an increase (in microns) of 20.93 in BMO-MRW, 4.26 in RNFL, and 7.85 in MT in CCS group (*P* < 0.001), and 17.7, 3.73, and 5.65, respectively, in FLACS group (*P* < 0.001). Six months after surgery, there was an increase of 12.53 in BMO-MRW, 1.42 in RNFL, and 4.72 in MT in CCS group (*P* < 0.001), and 13.7, 1.88, and 4.14, respectively, in FLACS group (*P* < 0.001). The postoperative changes in CCS group were similar to those in FLACS group.

**Conclusion:**

CCS as well as FLACS result in a slight increase in BMO-MRW, RNFL, and MT values one month and six months after surgery. Neither CCS nor FLACS lead to a deterioration in the parameters that define the structure of the optic nerve head and the macula. These results suggest that FLACS is as safe as CCS regarding the optic nerve head and the macula in normal eyes.

## 1. Introduction

Glaucoma is a chronic disease that causes progressive damage to the optic nerve head (ONH) and can lead to blindness. It is more frequent in elder people–6% of the population over 70 years old suffer from this disease–and therefore, it often coexists with cataracts [1]. For many decades, phacoemulsification has been the most commonly used technique for cataract surgery, both in patients without any ocular disease and also in those with glaucoma. Nowadays, many surgeons prefer femtosecond laser-assisted cataract surgery (FLACS) due to its advantages over conventional cataract surgery (CCS) such as increased accuracy and reproducibility, reduced endothelial cell loss, and reduced effective phacoemulsification time [2–5]. Despite the scarcity of evidence regarding its effects on the ONH, the number of publications stating that FLACS is especially useful in certain conditions related to glaucoma keeps increasing [6, 7]. These conditions include pseudoexfoliation, narrow anterior chamber, angle closure, nanophthalmos, and phacomorphic glaucoma [6–11].

FLACS requires the application of a suction device to stabilize the eyeball which causes an increase in intraocular pressure (IOP). This poses potential risks, especially for patients with glaucoma. Some studies have proved that FLACS does not lead to a greater macular thickening than CCS [12–16]. Regarding the ONH structure, it has not been established if FLACS is as safe as CCS.

Optical coherence tomography (OCT) is currently used for routine evaluation of the macula and the ONH, since it is a fast and accurate method capable of detecting structural damage and also changes over time [17, 18]. It provides measurements of the two parameters that have classically defined the structural status of the macula and the ONH–macular thickness (MT) and peripapillary retinal nerve fiber layer (RNFL). Recently, the Bruch's membrane opening was found to be the true anatomical border of the optic disc, and this led to the appearance of a new OCT parameter called Bruch's membrane opening minimum rim width (BMO-MRW) [19, 20]. The BMO-MRW represents an accurate measurement of the neuroretinal rim [19], and thus, it has become a key parameter to detect structural damage in the ONH. Some studies have shown its ability to detect glaucomatous damage earlier than RNFL [21, 22]. Due to its sensitivity to slight variations, this new parameter could be useful for the detection of changes in the ONH caused by the intraoperative IOP increase that occurs during cataract surgery, both in CCS and FLACS.

The aim of this study was to evaluate and compare the changes in BMO-MRW, RNFL, and MT one month and six months after cataract surgery, in CCS and FLACS.

## 2. Methods

### 2.1. Study Design and Patients

This nonrandomized prospective case series included patients scheduled for FLACS (FLACS group) or conventional cataract surgery (CCS group) in Vithas Eurocanarias Instituto Oftalmológico, Las Palmas de Gran Canaria, Spain. Written informed consent was obtained from each subject after receiving a full explanation of the procedure and the nature of the study. The study was approved by the local ethics committee and was performed in compliance with the tenets of the Declaration of Helsinki.

Inclusion criteria for both groups were age over 45 years old, cortical nuclear sclerotic grade II-III cataracts according to the Lens Opacities Classification System (LOCS) III classification, and axial length between 21.0 and 25.5 millimetres (mm). Exclusion criteria were previous ocular surgery, history of any systemic or ocular diseases (e.g., glaucoma, ocular hypertension, age related macular degeneration), any condition that could alter OCT results (e.g., peripapillary atrophy, difficulties in fixation, corneal opacities), low quality OCT images (image quality under 15), spherical equivalent refraction over 3 diopters, and intraoperative or postoperative complications. After a detailed explanation of both techniques, patients chose which surgical option they preferred; those who decided to have cataract surgery with femtosecond laser assistance comprised the FLACS group and the rest comprised the CCS group.

### 2.2. Preoperative and Postoperative Evaluations

All patients underwent comprehensive slit lamp examination before and one day, one week, one month, and six months after surgery. Preoperative tests included biometry (IOL Master® 700, Carl Zeiss Meditec, Jena, Germany) and Scheimpflug corneal topography (Pentacam Scheimpflug Image System, Oculus Inc. Wetzlar, Germany). Corrected and uncorrected visual acuity was recorded. Prior to surgery spectral-domain OCT (Spectralis–Glaucoma Module Premium Edition, Heidelberg Engineering, Carlsbad, CA, USA), circle and radial scans were acquired to provide RNFL and BMO-MRW measurements, respectively, as well as horizontal scans to provide MT measurements. Circle and radial scans were centered on the BMO, and all scan types were aligned according to the fovea-to-BMO-center (FoBMOC) axis using the automated anatomical positioning system (APS) scan feature. The APS-based scans were repeated one month and six months after surgery using the automatic “follow-up” feature in order to provide BMO-MRW, RNFL, and MT measurements. IOP measured by Goldmann applanation tonometry was also assessed preoperatively, one month and six months after surgery.

### 2.3. Surgical Technique

All surgeries were performed by the same surgeon (H. C.), under topical anaesthesia and using the Centurion phacoemulsification system (Alcon Laboratories, Inc.). In the FLACS group, two clear corneal incisions, the capsulorhexis, and a combined radial and cylinder pattern lens fragmentation were performed with the LenSx laser system (Alcon Laboratories, Inc.), using the Contact lens SoftFit™ interface. The soft hydrogel contact lens matches the corneal curvature and reduces the pressure needed to fix the eyeball, minimizing the IOP increase. A 2.2 mm corneal incision was fixated at 135°, and a 1.0 mm corneal incision was fixated at 45°; capsulorhexis diameter was 5 mm. In the CCS group, a 2.2 mm incision at 135° and a 1.0 mm incision at 45° were performed by the surgeon with a keratome, capsulorhexis was created with an intended diameter of 5 mm, and vertical phaco-chop technique was used. Both in FLACS and CCS groups, a single-piece hydrophobic acrylic aspheric intraocular lens (IOL) was inserted in the bag through the 2.2 mm incision. The two types of IOL used were AcrySof IQ SN60WF (monofocal IOL) and AcrySof IQ PanOptix (multifocal IOL). In both groups, the postoperative treatment consisted of topical application of antibiotic (ofloxacin 3 mg/ml eyedrops 4 times a day for 10 days), nonsteroidal anti-inflammatory drugs (bromfenac 0.9 mg/mL eyedrops twice a day for 1 month), steroid (dexamethasone 1 mg/ml eyedrops 4 times a day for 10 days, twice a day for 7 days, once a day for 7 days), and artificial tears.

### 2.4. Statistical Analysis

Statistical analyses were performed using R Core Team 2020 version 4.0.2 (R: A language and environment for statistical computing, R Foundation for Statistical Computing, Vienna, Austria). The Kolmogorov–Smirnov test was used to determine whether the variables were normally distributed. Mean and standard deviation (SD) of all parameters were calculated. For the comparisons of continuous variables between groups, such as age and axial length, *t* tests were used. For the analysis of categorical variables, such as the female-male ratio and the monofocal-multifocal ratio in each group, the Fisher exact test was used. Linear regression analysis for paired data was used to compare the 1-month postoperative RNFL, BMO-MRW, and MT values with the preoperative values, as well as to compare the 6-month postoperative values with the preoperative values, both in CCS group and FLACS group. Linear regression analysis was also used to determine the differences between the changes in the three OCT parameters in CCS group and those in FLACS group. In order to compare the postoperative behaviour in FLACS group with that of CCS group, linear regression analysis with interaction was used. A *P* value less than 0.05 was considered statistically significant.

## 3. Results

A total of 146 eyes of 146 patients were included in this study. Of these, 65 eyes underwent CCS, and 81 underwent FLACS. The difference between the mean age of patients in CCS group and FLACS group was not statistically significant. Axial length (AL), preoperative optical anterior chamber depth (ACD), preoperative IOP, and the female-male ratio were similar in both groups, as shown in Table 1. There were statistically significant differences in the monofocal-multifocal IOL ratio between groups; 47 eyes (72%) in CCS group had a monofocal IOL implanted while this occurred only in 28 eyes (34.6%) in FLACS group (*P* < 0.001). Preoperative IOP was 15.51 ± 2.62 in CCS group and 16.19 ± 3.27 in FLACS group; one month after surgery, it was 13.09 ± 2.61 in CCS group and 13.60 ± 2.78 in FLACS group, and six months after surgery, it was 13.12 ± 2.60 in CCS group and 13.26 ± 2.64 in FLACS group. This postoperative IOP reduction was statistically significant in both groups (*P* < 0.001), as shown in Table 2. Preoperative BMO-MRW, RNFL, and MT were 329.54 ± 57.78, 102.14 ± 9.33, and 278.78 ± 17.92 in CCS group, and 324.67 ± 50.72, 99.53 ± 10.76, and 269.15 ± 25.19 in FLACS group.

### 3.1. Changes in BMO-MRW, RNFL, and MT One Month and Six Months after Surgery

**Figure 1:**
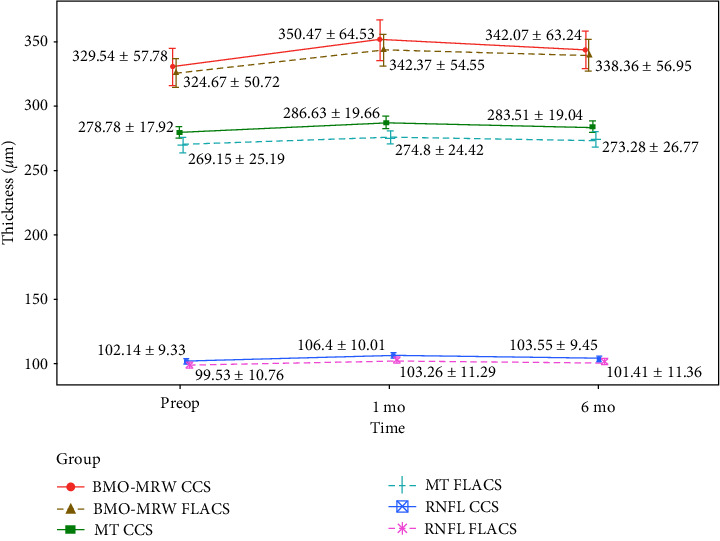
BMO-MRW, RNFL and MT at all visits in CCS and FLACS groups. BMO-MRW = Bruch's membrane opening minimum rim width. RNFL = peripapillary retinal nerve fiber layer. MT = macular thickness. CCS = Conventional cataract surgery. FLACS = femtosecond laser-assisted cataract surgery. Preop = Preoperative. 1 mo = 1 month potoperative. 6 mo = 6 months postoperative. Data are reported as mean ± SD.

After surgery, both in CCS group and FLACS group, there was a slight increase in BMO-MRW, RNFL, and MT (Figure 1). This increase was more pronounced one month after surgery than six months after surgery and showed a tendency to return to baseline values without reaching them after six months. The difference between one-month postoperative values and preoperative values was statistically significant for the three parameters in both groups (*P* < 0.001), as was also the difference between six-month postoperative values and preoperative values (Table 3).

### 3.2. Comparison between CCS Group and FLACS Group

After surgery, the behaviour of the BMO-MRW, RNFL, and MT was similar in CCS group and in FLACS group. Regarding BMO-MRW and RNFL, the difference in the mean ± SD between CCS group and FLACS group was not statistically significant at any of the follow-up visits. On the contrary, MT was slightly thinner in FLACS group than in CCS group at the three time points (*P* < 0.05), as shown in Table 4.

Figure 1 shows the postoperative behaviour of the three parameters. Regarding BMO-MRW and RNFL, the baseline and the postoperative behaviour were similar in both groups. Regarding MT, although there was a slight but statistically significant difference in the baseline values, the behaviour was similar during the follow-up in both groups.

## 4. Discussion

The aim of this study was to evaluate and compare the changes in BMO-MRW, RNFL, and MT one month and six months after cataract surgery, in CCS and FLACS. Some studies have shown that FLACS does not lead to more macular thickening than CCS [12–16], but there is a paucity of evidence regarding the effects of CCS and FLACS on the ONH [23, 24]. In the present study, the postoperative behaviour of BMO-MRW, RNFL, and MT was very similar in both groups. A slight increase in the three parameters was observed one month after surgery, and posteriorly, there was a tendency towards returning to baseline values without reaching them after six months. Neither CCS nor FLACS caused changes indicating deterioration in the structural status of the ONH.

During CCS, there is a transitory but significant rise in IOP. FLACS implies an additional increase in IOP that occurs during the application of the suction ring used to stabilize the eye [8, 25]. Thus far, it is unknown if this increase in IOP is long or intense enough to pose some risk to the ONH in healthy or in glaucomatous eyes. Regarding CCS, the results of the study by Zhao et al. [26] showed IOP fluctuations from 13 ± 4.7 to 96 ± 6.2 mm Hg during the main steps of phacoemulsification. According to a study by Vasavada et al. [27], during phacoemulsification, the maximum IOP ranged from 69 ± 3.0 to 85 ± 1.2 mm Hg depending on the fluidic parameters. Research in cadaver eyes by Khng et al. [28] found IOP rises over 60 mm Hg. With respect to FLACS, several studies have assessed the changes in IOP caused by the different femtosecond platforms. The estimated IOP increase for Victus, Ziemer LDV Z8, and Catalys is 42, 30, and 18.5 mm Hg, respectively [25, 29–32]. A literature review on the LenSx platform showed an increase in 16 mm Hg during 1–2 minutes and concluded that patients with glaucoma may not be at risk [8]. Darian-Smith et al. [33] investigated the IOP changes in glaucomatous and nonglaucomatous eyes using a Catalys platform and found that the increase in IOP was greater in eyes with glaucoma (17.04 vs. 14.01 mm Hg). They concluded that this IOP rise was well-tolerated short term, and that long-term implications were unknown. Certainly, the long-term effect of those intraoperative IOP spikes on the ONH is unknown, but the results of several studies [23, 33] and those of our own suggest that in the mid-term, no damage to RNFL or BMO-MRW can be detected.

Cataract surgery causes variations in IOP intraoperatively, as previously discussed, but also postoperatively. It has been demonstrated that after phacoemulsification, there is a reduction in IOP of variable degree, depending on factors such as preoperative IOP, preoperative anterior chamber depth, lens thickness, and lens position, both in healthy and in glaucomatous eyes [34–38]. A study by Coh et al. [39] assessed the IOP change 4 months after CCS in nonglaucomatous and glaucomatous eyes and found a decrease in IOP of 2.80 ± 3.83 mm Hg from the preoperative mean of 14.73 ± 2.89 mm Hg in nonglaucomatous eyes–similar to the IOP decrease in our study–and an IOP decrease of 2.66 ± 2.07 mm Hg from the preoperative mean of 14.86 ± 2.97 mm Hg in glaucomatous eyes. Glaucoma surgery usually causes drastic reductions in IOP, and this can lead to changes in the appearance of the optic disc called “optic disc cupping reversal” [40]. After cataract surgery, the decrease in the IOP may have some expanding effect on the neuroretinal rim similar to that observed after glaucoma surgery, though less marked. This might be the cause of the increase in BMO-MRW found after CCS and FLACS in the present study.

In addition to the changes in OCT measurements of BMO-MRW, RNFL, and MT that can occur due to variations in IOP, changes in these measurements caused by the implantation of an IOL must be taken into account. The type of IOL seems to have an influence on the acquisition of the images by the OCT device. A study by Celik et al. [41] showed small changes in MT and RNFL after CCS with monofocal IOL implantation. Specifically, MT increased from 247.9 ± 17.6 to 249.0 ± 17.8 and RNFL increased from 97.4 ± 5.4 to 101.7 ± 5.6 one month after surgery, *P*=0.029 and *P* < 0.001, respectively. Hence, they concluded that new baseline measurements should be obtained after surgery. A few studies have corroborated that after CCS with monofocal IOL implantation, there is a slight increase in MT and RNFL [42–45]. García-Bella et al. [46] found an increase in RNFL three months after CCS with multifocal IOLs (96.77 vs. 99.55). To the best of our knowledge, only one study [47] has compared the effects of monofocal vs. multifocal IOL implantation on the change in OCT measurements. It assessed only one parameter, the RNFL, and found an increase in OCT measurements after CCS in both groups, but its magnitude was greater in the case of multifocal IOL implantation. Sánchez-Sánchez et al. [23] assessed MT and RNFL preoperatively and three months after cataract surgery with multifocal IOL implantation, both in CCS and FLACS groups. In the CCS group, preoperative MT and postoperative MT were 260.44 ± 22.23 and 264.61 ± 22.02 (*P* < 0.001); the difference between preoperative and postoperative RNFL was not statistically significant. In the FLACS group, postoperative values were significantly thicker for MT (259.23 ± 19.70 vs. 264.57 ± 20.80, *P* < 0.001) and for RNFL (90.22 ± 8.99 vs. 91.01 ± 9.90, *P*=0.019). In our study, the OCT was performed at three time points (before surgery, one month after surgery, and six months after surgery), and different types of IOL were used; however, our results are in accordance with the latter.

The usefulness of RNFL measurements for the diagnosis and follow-up of glaucoma patients has been well stablished [17, 18]. Recently, the BMO-MRW has also become a key parameter in glaucoma management. It has been proved that this measurement of the optic disc cupping shows high reproducibility and diagnostic ability in glaucomatous neuropathies [19]. It is especially useful for the detection of incipient or slight damage in the ONH, since BMO-MRW parameters show a strong capability to differentiate between mild glaucoma and control eyes [48]. Slight deteriorations in the ONH can be detected earlier measuring BMO-MRW compared to RNFL; in fact, considerable BMO-MRW thinning precedes RNFL thinning in the course of glaucoma [21]. A study by Gardiner et al. [22] concluded that MRW may be more sensitive for early detection of glaucomatous damage whereas RNFL may be preferable for monitoring change. In addition to this, BMO-MRW analysis provides significantly greater specificity than RNFL in especially complex cases like tilted disc with low and moderate myopia [49]. This may be related with the findings of a study by Chauhan et al. [19], who stated that BMO-MRW may be more sensitive to ONH conformational changes that precede but do not directly correlate to RNFL loss. Some studies have shown that acute increases in IOP cause significant changes in BMO-MRW [50, 51], and it has also been suggested that glaucoma subjects are more susceptible to these IOP increases compared to normal subjects [52]. In spite of the intraoperative IOP increases that occur during CCS and FLACS, in the present study, there was not a decrease in BMO-MRW following cataract surgery. It would be ideal, but not feasible, to assess them in real time during surgery. We could speculate that even though changes in the configuration of the nerve do occur during IOP spikes [53], like that caused by suction during FLACS, the results of our study suggest that they are only transient and do not last overtime in healthy eyes. Consequently, CCS and FLACS seem to be equally safe for the ONH structure in healthy eyes. However, these results may be different in glaucomatous eyes.

The present study is the first to assess and compare the changes caused by CCS and FLACS not only in MT and RNFL but also in BMO-MRW. Moreover, so far, there are no reports demonstrating permanent changes in these OCT parameters with a longer follow-up than that in our study. However, this study has certain limitations, such as the relatively small sample size, the use of two different types of IOL with different distribution in the groups, and the fact that each patient decided whether to undergo CCS or FLACS. Furthermore, since the slight thickening observed in BMO-MRW, RNFL, and MT after surgery tended to decrease over time, a longer follow-up would be desirable to determine whether the values of the three OCT parameters eventually return to baseline or if the slight increase remains stable after six months. Our findings do recommend that after CCS and FLACS, new baseline images should be acquired for accurate follow up of ONH structure, better at six months than earlier.

In conclusion, this study showed that BMO-MRW, RNFL, and MT behave in a similar manner after CCS or FLACS. In both groups the three parameters increased slightly postoperatively; this increase was less pronounced 6 months after surgery than one month after surgery. CCS and FLACS seem to be equally safe regarding the structure of the ONH and the macula, as measured by SD-OCT. Further studies are necessary to assess if there are different results in glaucomatous eyes.

## Figures and Tables

**Table 1:** Demographic data and characteristics of enrolled patients.

Parameters	CCS group	FLACS group	*P* value
Age (y)	68.12 ± 9.37	66.23 ± 7.3	0.186
Female-male ratio, *n* (%)	40 : 25 (61.5%:38.5%)	52 : 29 (64.2%:35.8%)	0.863
Monofocal-multifocal IOL ratio, *n* (%)	47 : 18 (72%:28%)	28 : 53 (34.6%:65, 4%)	<0.001
AL (mm)	23.58 ± 0.81	23.69 ± 1.12	0.514
ACD (mm)	3.11 ± 0.36	3.20 ± 0.41	0.142
Preoperative IOP (mm hg)	15.51 ± 2.62	16.19 ± 3.27	0.141

IOL = intraocular lens. AL = axial length. ACD = anterior chamber depth. IOP = intraocular pressure. Data are reported as mean ± SD.

**Table 2:** Difference between postoperative and preoperative IOP in CCS and FLACS groups.

		1 mo-preop		6 mo-preop	
Parameter	Group	ΔMean (95% CI)	*P* value	ΔMean (95% CI)	*P* value

IOP (mm hg)	CCS	−2.42 (−2.98–−1.85)	<0.001	−2.38 (−2.95–−1.82)	<0.001
	FLACS	−2.58 (−3.09–−2.07)	<0.001	−2.93 (−3.44–−2.42)	<0.001

IOP = intraocular pressure. Preop = Preoperative. 1 mo = 1 month potoperative. 6 mo = 6 months postoperative. Data are reported as the difference in mean values (ΔMean) with its respective 95% confidence interval (95% CI).

**Table 3:** Difference between postoperative and preoperative values of BMO-MRW, RNFL and MT in CCS and FLACS groups.

		1 mo-preop		6 mo-preop	
Parameter	Group	ΔMean (95% CI)	*P* value	ΔMean (95% CI)	*P* value

BMO-MRW	CCS	20.93 (18.06–23.8)	<0.001	12.53 (9.66–15.4)	<0.001
	FLACS	17.7 (15.13–20.27)	<0.001	13.7 (11.13–16.27)	<0.001
RNFL	CCS	4.26 (3.65–4.87)	<0.001	1.42 (0.81–2.02)	<0.001
	FLACS	3.73 (3.18–4.27)	<0.001	1.88 (1.33–2.42)	<0.001
MT	CCS	7.85 (5.63–10.07)	<0.001	4.72 (2.5–6.94)	<0.001
	FLACS	5.65 (3.67–7.64)	<0.001	4.14 (2.15–6.12)	<0.001

Preop = preoperative. 1 mo = 1 month potoperative. 6 mo = 6 months postoperative. Data are reported as the difference in mean values (ΔMean) with its respective 95% confidence interval (95% CI).

**Table 4:** Difference in BMO-MRW, RNFL and MT between CCS and FLACS groups in the three follow-up visits.

	CCS preop–FLACS preop		CCS 1 mo–FLACS 1 mo		CCS 6 mo–FLACS 6 mo	
Parameter	ΔMean (95% CI)	*P* value	ΔMean (95% CI)	*P* value	ΔMean (95% CI)	*P* value
BMO-MRW	−4.87 (−23.58–13.84)	0.61	−8.09 (−26.81–10.62)	0.397	−3.71 (−22.42–15)	0.698
RNFL	−2.61 (−6.01–0.79)	0.133	−3.14 (−6.54–0.26)	0.07	−2.15 (−5.54–1.25)	0.216
MT	−9.64 (−17.02–−2.25)	0.011	−11.83 (−19.21–−4.44)	0.002	−10.22 (−17.61–−2.84)	0.007

Preop = preoperative. 1 mo = 1 month potoperative. 6 mo = 6 months postoperative. Data are reported as the difference in mean values (ΔMean) with its respective 95% confidence interval (95% CI).

## Data Availability

The data used to support the findings of this study are available from the corresponding author upon request.
